# Overlapping worlds of art and plastic surgery: developing a concept model and its implications in surgical education

**DOI:** 10.1007/s44186-022-00089-y

**Published:** 2022-12-10

**Authors:** Audrey Nguyen, Dawn Duong, Patricia O’Sullivan

**Affiliations:** 1 Department of Surgery, Division of Plastic and Reconstructive Surgery, University of California San Francisco (UCSF), 505 Parnassus Ave, Suite M-593, San Francisco, CA 94143 USA; 2 School of Medicine, Departments of Medicine and Surgery, University of California San Francisco, San Francisco, USA

**Keywords:** Education, Plastic surgery, Arts and humanities, Visual arts, Curriculum

## Abstract

**Purpose:**

Editorials speculate on the relationship between art and plastic surgery, and studies of limited art education in surgical training show intriguing benefits. Identifying the shared concepts and skills in art and plastic surgery could advance incorporating artistic skills and concepts into plastic surgery training and curriculum.

**Methods:**

Using a grounded theory approach, we interviewed plastic surgeons and formally trained or self-identified artists and then analyzed the transcripts. During the process, we used a constant comparison approach while coding along with data collection. The team developed the codebook from initial transcripts; 2 members coded each transcript. We reconciled codes and summarized codes into themes based on discussion among the team.

**Results:**

15 plastic surgeons aged 36–80 years and 16 artists aged 19–62 years were interviewed. We then developed a concept model, “Ways of Making,” to illustrate the shared aspects of the artistic and surgical process through their *ways of doing, knowing, seeing, and thinking.* Both plastic surgeons and artists recognized that strong technical foundational skills are key to developing competency. Both groups spoke about the *Elements of Art* and *Principles of Design*, though artists know this formally. Artists and plastic surgeons shared that awareness to one’s surroundings or to human features facilitates identifying problems or ideas. They described how technical skills, manual dexterity, and three-dimensional thinking can be taught and nurtured. Both groups also recognized that creativity played a major role in their work. While creativity was seen as innate, participants can learn to be innovative through critical thinking.

**Conclusion:**

This study provides a model for how plastic surgery and art overlap using data from interviews. Though there are differences between the two fields, the *ways of doing, knowing, seeing, and thinking* are key components of the artistic and surgical processes. Identifying the shared concepts and skills in art and plastic surgery could help enhance curricula seeking to incorporate artistic skills and concepts into plastic surgery training.

## Introduction

The relationship between art and plastic surgery has interested plastic surgeons for almost fifty years [1–6]. Published studies about this relationship are mostly editorial in nature, but limited art education in surgical training has shown intriguing benefits. Surgeons have published opinions in editorials and correspondence about art and plastic surgery. These opinions describe the importance of understanding beauty, grace, and esthetics [6, 7], how knowledge of art helps with plastic surgeons’ three-dimensional visual-spatial skills [8], or how the fundamental difference is that art is a form of self-expression whereas plastic surgery is a science that “seeks to uncover knowledge (and arguably beauty)” [2]. In one survey study of 26 plastic surgery trainees in England, who were asked whether they felt that artistic skills were relevant in plastic surgery; 58% agreed [7]. Notably, many of the published comments were about the ability to draw, and one of the trainees who disagreed wrote, *“Just because you can draw does not mean you can operate competently.”*

However, art goes beyond the act of drawing. A study from the Giesel School of Medicine in Dartmouth, New Hampshire, introduced art in a different way [9]. The authors showed a painting titled, *In Hospital (The Operating Room),* by Bernard Perlin (1918–2014) to surgical trainees and academic surgeons and asked them to describe what they saw. The results were interesting; the trainees described the painting in clinical terms and focused on anatomy and technique while the surgeons focused on the primary surgeon and his role in the painting. The authors concluded that the understanding of perspective and mature clinical gaze can be established through observations and analyses of works of art [9]. The Pennsylvania Academy of Fine Arts offers art courses to medical students and personnel with the goal of “improving visual literacy” and observational skills [10]. Casey Lesser, Creativity Editor of Artsy, an online platform for collecting and discovering art, writes that medical students have been required to take art classes to help improve communication skills, perspective, and even, compassion [11]. Though anecdotal, these opinion pieces approach an appealing idea—that an arts education is beneficial to surgical training.

Exploring the shared core concepts and skills in the fields of art and plastic surgery could inform plastic surgery training programs about what aspects of artistic skills and concepts can be applied to training. Art education courses and artistic skill are not a requirement of plastic surgery trainees, nor are they assessed in the current model of competency based medical education (CBME), which is set as the structure for assessing performance in United States Plastic Surgery Programs accredited by the Accreditation Council of Graduate Medical Education (ACGME) [12, 13]. However, plastic surgery programs aspire to educate residents who can demonstrate skills resulting in aesthetically pleasing results, who are experts in human anatomy, and who have the observational and surgical skills to design and complete a successful surgery [14]. The goal of this study is to describe the core concepts and skills that art and plastic surgery share to provide the language and a new framework necessary to enrich the curriculum and competencies for plastic surgery trainees. The findings may provide guidance for educating surgeons by incorporating the skills and practices of the related field of visual arts.

## Methods

We conducted interviews and qualitative analyses of the interview transcripts using grounded theory—a qualitative research method used by investigators to generate a theory that explains a process or action based on “data from participants who have experienced the process” [15]. We identified that this approach was useful since we were unsure how concepts from artists and surgeons would overlap and because there is sparse literature and underlying theories related to this topic. There are editorial and opinion pieces in the plastic surgery literature that suggest a relationship between the two fields that improves plastic surgery skill as previously discussed [1–8]. However, there is a lack of a model that describes how art and plastic surgery are related. We chose interviews as a data collection method because they provide rich data for the development of a model for this grounded theory study. An interview “attempts to understand the world from the subjects’ point of view, to unfold the meaning of their experience, to uncover their lived world” [16]. The grounded theory methodology allows us to identify and understand a way in which the concepts that are important to both artists and plastic surgeons can be interrelated.

We recruited practicing plastic surgeons and practicing artists to interview for this study. Plastic surgeons were eligible to participate if they were board-certified or board-eligible. Artists were eligible if they identified as an artist for their primary occupation or if they had formal visual arts training and education. Many of the recruited surgeons and artists were considered experts in their field. They were considered experts based on academic or social status, years in practice, and productivity. The presented sample size is typical of studies using grounded theory approaches, as the purpose of the study is to achieve an understanding and saturate the underlying theory model [15]. We sampled until we believed information sufficiency had been obtained, which fell in the range of our goal sample size [15].

Using purposeful sampling [17], we recruited academic and community plastic surgeons who represent all subspecialities within the field using the network of the principal investigator, AN. She started with her local network of academic plastic surgeons whom she trained with at UCSF and also engaged in artistic activities (sculpture, photography, digital art). She also interviewed plastic surgeons who were considered experts and with whom she had a relationship. AN also approached plastic surgeons via email who have presented at conferences and expressed an interest in the visual arts based on their lecture. AN contacted all plastic surgeons personally and did not use any other recruitment methods. AN also knew some artists and used a snowball sampling approach by asking artist participants to recommend other participants. Like the plastic surgeons, we selected the participants based on their background and specialty to include a representative sample of visual artists who use different media and/or are in varying types of practices. We also included artists who were educators, as this was comparable to plastic surgeon educators in academia. This study was approved by the UCSF Institutional Review Board (IRB).

### Data collection

*Interview Guide*: We developed a preliminary interview guide based on literature review and the experiential knowledge of AN. We used two framing questions. First, we asked participants to describe in detail the core concepts, principles, and competencies that they feel are important to their field, including those directly taught in training and those developed via extramural learning. Second, we asked if there were specific skills that they developed or were developing to become a competent or master surgeon or artist. During the interviews, we probed participants to further explore the themes that the research team was identifying from prior interviews. We used this semi-structured approach since it “can help ensure the comparability of data across individuals, times, settings and researchers, and are particularly useful in answering questions that deal with differences between people or settings” [17].

*Interviews*: We conducted single, semi-structured interviews that lasted 60–120 min. Each interviewee provided demographic data such as age, gender, and specialty. The initial five interviews were performed in-person and recorded. However, at the start of the COVID-19 pandemic, we switched to Zoom interviews for safety. These interviews were audio and video recorded on Zoom. The recordings allowed for verbatim transcription of the interview through artificial intelligence (Rev.com). Review of the conversation and video by the investigators and transcriptionist permitted checks for accuracy and extraction of narratives, themes, and quotations. Each interviewee received a $75 Amazon gift card for their participation.

### Analysis

The research team developed a codebook using in vivo codes from the initial transcripts. The team used a constant comparison approach and met throughout the data collection, coding, and analysis processes. Each member’s views were discussed and considered as the analysis progressed. AN is a plastic surgeon with some experience in art courses but limited formal training, and she has illustrated plastic surgery articles and texts. She is completing a Master of Art in Education. AN completed the plastic surgery interviews. DD has also taken art classes and has been involved in other qualitative research studies. DD was comfortable talking with educators and performing interviews. AN and DD did the artist interviews. PO’S provided expertise in qualitative analysis. All team members served to add perspective and to challenge AN to clarify how her perspective influences the interpretation of the results.

We used the software Dedoose (SocioCultural Research Consultants, LLC, Los Angeles, CA) to code and to organize the analysis of the interview transcripts. Using Dedoose, each transcript was coded by a single researcher who was blinded to the other researcher’s proposed codes. The researchers then reviewed each assigned code and came to an agreement on the codes through discussion and reference to the codebook if the codes were divergent. We memoed throughout the interview and coding process. We then summarized our codes into themes based on discussion among the team.

## Results

### Demographic information

A total of 31 in-depth interviews were conducted with 15 plastic surgeons and 16 artists (Table 1). The plastic surgeons ranged from 36 to 80 years of age, and the artists ranged from 19 to 60 years. In both groups, most participants identified as White. There were three artists who identified as Latinx. There was an even spread between gender in the artist group, with plastic surgery having slightly more participants who identified as men (*n* = 9) than women. Both groups interviewed represented a variety of subspecialties in their respective fields. Eight of the plastic surgeons were educators at a university or university-affiliate, and four artists were educators at the junior high, high school or college level. While we interviewed this large number of participants, we felt information sufficiency had been reached in both groups around the 10th interview.

**Table 1:** Demographic and practice information about the two groups of participants, plastic surgeon and artist

	Plastic surgeon (*N* = 15)	Artist (*N* = 16)
**Age, years**	36–80	19–60
**Gender, N**		
Male	9	8
Female	6	8
**Race, N**		
White	13	12
Asian	2	1
Latinx	0	3
**Specialty, N**	Cosmetic Surgery, 5	Multimedia*, 9
	Microsurgery, 5	Painting, 3
	Craniofacial Surgery, 3	Drawing, 1
	Hand Surgery, 2	Sculpture, 1
	General Reconstruction Only, 1	Graphic Design, 1
Practice Type	Academic, 8	Educator, 4
	Private Practice, 7	Personal Business, 12

^*^More than 1 type of media (i.e., ceramics, photography, drawing, and painting)

### Concept model

**Fig. 1:**
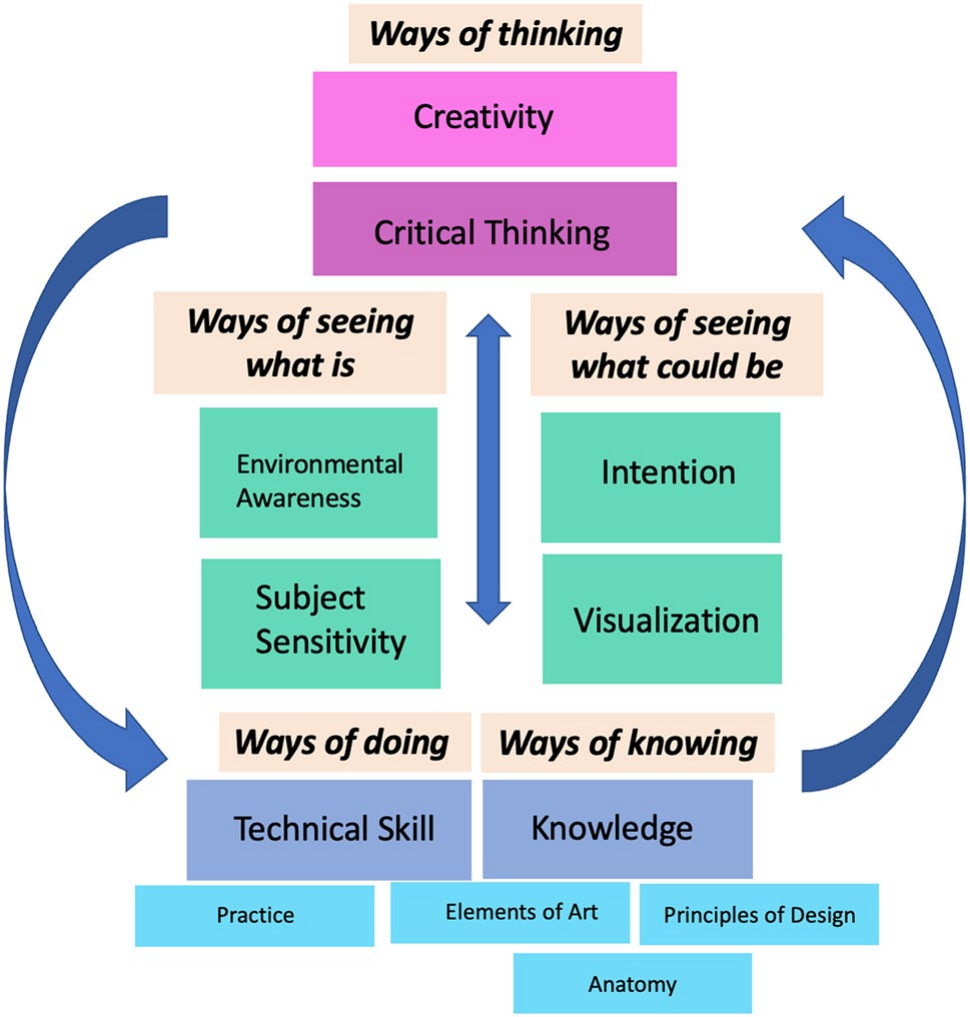
Concept Model of *Ways of Making* – a framework that demonstrates the artistic and plastic surgery process

The concept model that we developed based on our intensive interviews revealed a framework for understanding how art and plastic surgery overlap that we have titled, “Ways of Making” (Fig. 1). “Making” referred to the finished product or work. For visual artists, this was their artwork. For plastic surgeons, this was the surgery and its results. The model was organized into five interrelated categories: *ways of knowing, ways of doing, ways of seeing what is, ways of seeing what could be, and ways of thinking*. Table 2 highlights quotes supporting each concept.

**Table 2:** Exemplary quotes from artists and plastic surgeons as it relates to the concept model by concept. A refers to artists, and PS refers to plastic surgeon

	Artist	Plastic surgeon
Ways of doing Technical Skill: the use of tools, methods, and techniques	“So, I think that learning how to draw from life is very important to be able to see and translate what you see in reality, the physical reality, and that's why figure drawing is important. The anatomy is important, because it's a huge instructional thing about drawing the world. Learning how to draw from what you see, you learn the abstract relationships between objects, between form.” (A6)	“Dexterity. I'm watching people sew, watching people do stuff.” (PS5) “So I think that that can be taught, in how you motion, how you move, how you handle things, the instruments that you use, and why or not.” (PS6)
Ways of knowing Elements of art: building blocks used by artists to create a work of art and include line, shape, form, color, value, space, and texture Principles of design: the way artists use the *elements of art* in their work: balance, emphasis, movement, pattern, repetition, proportion, rhythm, variety, and unity Anatomy: the study and knowledge of the human body	“The first one is value. And if you take white at one end and black at the other end, and then you have nine shades in between or eight shades in between, you establish what are called values…And whenever we paint something, it's a matter of breaking it down into shapes.” (A13) “Classes that teach you how to draw a human form, classes that teach you about color, about the principle of design, classes that teach you how to work with your hands, and classes that teach you how to work computer programs. All classes that will give you your core values and what people are expecting from you in terms of your knowledge of art.” (A15) “In anatomy class for instance, there was a lot of drawing live skeletons and that kind of stuff. And one of my professors was always over the top, so in that particular class, we would go and unwrap cadavers and we would draw from that. And I actually found that very fascinating.” (A16) “So, if I'm dealing with a human body, and I understand those things, I understand then physically, the relationship of one part to the next, and how it fits into a volume. And that to me seems like that would be an incredibly valuable for somebody who was dealing with human bodies.” (A11)	“So, I think I improved my anatomy a ton and learning about art and art techniques made me look at things differently. All of a sudden, I realized things were there that I never knew were there, like shadows and different light intensities and shades, and I didn’t even know what I didn’t know, and it was nice to learn that and I think it helped me understand anatomy better. It helped me understand, for instance, cheekbones and the points of shining on light and lips and ears and things like that. So, when I was learning how to do it, it was very exciting for me because I thought I was getting better at surgery because I was learning this art knowledge.” (PS4) “Well again, the aesthetic eye is just it’s seeing any part of the human body and knowing what looks right or what looks wrong, what looks esthetic or not, what looks pretty, what looks symmetrical, what looks balanced, what looks proportional.” (PS7) “I think it's important that you have a basic understanding of anatomy, and principles, and basic principles of flap design, and stuff like that, and then when you see a problem, you can figure out how to apply that in a specific case that may be novel or may be a little different from the way something else was done.” (PS5) “So, I think a good plastic surgeon has a great knowledge of anatomy, has a creative mind and then has the technical skills to execute something difficult in rearranging tissue.” (PS9)
Ways of seeing what is Environmental Awareness: being in tune and attentive to one’s surroundings Subject Sensitivity: the ability to see the subtleties of the subject matter than an untrained eye would not	“Because even when I walk, I walk daily, I can't help but notice patterns and the sunlight coming through the trees and the shadows and all that. I see designs. I see that all the time.” (A16) “I think artistic people see things that normal people don’t look at or they are hypersensitive to esthetics. They have sensibilities that are very different from people who are outside of the arts. They’re sensitive to form and arts and principles of design. They notice the things in between the line. The message in between the lines.” (A8) “One of the things that happens to lots of people in drawing classes is, the teachers, they would be telling you, you have to look, you have to look, you have to look, and you have to see.” (A11)	"When I was trying to learn what is the earlobe shape, what does the back of the earlobe really look like? How do we inset the earlobe and a facelift, why, what is this angle the earlobe makes with the jawline. I would be in the elevator, and I would just stare at the back of people's heads.” (PS11) “And when people look at an ear, they don't look the way we look at an ear. They don’t dissect by helix, antihelix, scapha, inferior crus, concha, that kind of stuff. We do that, and so we’re seeing things that other people don’t see.” (PS1) “I see better and if I can see better and see more things, I think I can communicate with the patient better. If I'm looking at somebody and I just see two or three things versus 20 things, I think you having 20 things that I'm seeing makes my diagnosis better and my planning better.” (PS4)
Ways of seeing what could be Visualization: thinking ahead by picturing the final product and going through the process in one’s mind Intention: setting the goal that the artist or surgeon is trying to achieve	“I guess again, it depends on when I set out, I have certain goals of the level I want to get it to. For me, myself, that is I know what, in a way, the endpoint is going to be.” (A12) “You're thinking two steps ahead, or three steps ahead, because it's just natural, the way you're doing things.” (A16) “To train awareness, but also once you have awareness of something and language for describing it, then you can have command over it. And I think a lot of artists aren't able to put that into words maybe, but they are actively using it. So that's the difference, is intentionality.” (A8)	“Usually, I'm thinking about the end where I want to get and then thinking about steps on how to get there, but probably when I'm walking into the operating room, the literal image I have in my head is what the patient looks like when we're done.” (PS4) “You have to have the image in mind as well and you kind of know what you're aiming for.” (PS11) ""Okay, he's got all these little dots on the lip." I said, "Okay, so what are the goals of our operation?" He starts talking through the dots, and I broke it down and was like, "No, no, what are we really trying to accomplish here?"” (PS15)
Ways of thinking Critical Thinking: problem or puzzle solving along with finding creative solutions Creativity: pushing the limits or boundaries, thinking outside the box, and being original or new by going beyond the normal, average, or expected	“It's kind of like how engineers invent things to solve problems. We're artistic engineers. We're inventing designs to solve people's needs with marketing and things like that. A core value or something you would need to become a designer is the desire to solve a problem.” (A15) “When I say learning how to think, it's learning how to take your successes and your failures in particular and building on those. There's an old cliché that you can succeed without failure, and that's true. You go through life, and you fall on your face, and you get up, you learn from it, if you're smart you learn from it, and you build on that, and out of those mistakes, or that chaos comes something new, and these discoveries.” (A6) *“*I would say putting yourself in uncomfortable situations forces you to be creative because usually when something's uncomfortable, it’s sometimes unprecedented.” (A9)	*“*It's a surgical discipline where essentially no two operations are ever completely the same…you have a problem; the patient has a problem, and that problem needs to be solved and it's a way of using tissue to solve those problems creatively.” (PS12) “I think it is that you have a set of rules that create your framework, but you're not bound by them. You get to use that framework to solve a problem, and I think most… If you think about problem-solving in any field, whether it's engineering or literature, it's about being creative. In plastic surgery, it seems like it's all about problem-solving, so that's where that creativity comes in.” (P15) “Creativity is the ability to produce, see, think or in any other way, something new, different and astounding or surprising. That's creativity… the big C is where you're actually doing something that has intrinsically more value or more power to the person that's observing or to yourself” (PS7)_

The number following the letter is the participants anonymized research identifier

### Ways of knowing

Both groups shared *ways of knowing* which was based on shared knowledge of anatomy and fundamental art concepts: elements of art and principles of design. Artists learned the *elements of art* and the *principles of design* in foundational art courses, and those guided everything in making art*.* The *elements of art* are the building blocks used by artists to create a work of art and include line, shape, form, color, value, space, and texture [18]. The *principles of design* describe the way artists use the *elements of art* in their work: balance, emphasis, movement, pattern, repetition, proportion, rhythm, variety, and unity [19]. For plastic surgeons, understanding anatomy was one of the most important *ways of knowing*. Fundamental concepts for this field were knowing the blood flow and anatomy and replacing like with like.

Of these shared *ways of knowing,* the plastic surgeons understood and knew anatomy at an extremely detailed level. We defined anatomy as the study and knowledge of the human body. This was necessary for patient safety, and a core component of reconstruction was knowing the baseline anatomy to be altered. However, artists also studied anatomy to understand the underlying structures that are important for overlying contour, shape, and movement. Both groups recognized the importance of knowing surface anatomy—how the surfaces of something are shaped but what lies beneath. The other important way of knowing for plastic surgeons also came from the *elements of art* and *principles of design*, though informally and not through art classes. Plastic surgeons used many of the terms within the *elements of art* and *principles of design* as they described their work. Some of these terms included “color”, “symmetry”, “shadow,” “light intensity” and “balance.” These basic terms were used in addition to the scientific, medical, and anatomical terms to describe their process and surgical design.

### Ways of doing

Artists and plastic surgeons also shared *ways of doing*, which described how each group goes through the process of achieving a work—either a surgical case or a piece of artwork. This process was centered around technical skill, which referred to the use of tools, methods and techniques in each field. For visual artists, one of the most important skills was drawing and mark-making because it developed the artistic eye and hand–eye coordination. Other skills included mixing paint and understanding the chemistry, how to use the instruments and how to use one’s hands. For plastic surgeons, the basic skills were suturing, working and handling human tissue and its underlying structures, and using instruments while doing so. There were certainly differences in dissecting tissue and suturing versus preparing paint colors, canvases, and painting. However, it was clear from the interviews that manual dexterity and tactile sensation were integral to the success of both artists and plastic surgeons and was developed early in training. Both groups strongly believed that practice was a key to improvement in the field and many participants from both groups quoted that 10,000 h of practice was necessary.

The ways of knowing and ways of doing represented the foundations of the concept model, even though all aspects were interrelated. These foundations were important because once they were developed, they can be pushed and broken, allowing for freedom and creativity.

### Ways of seeing

The ways of knowing and doing were related to *ways of seeing*. This notion described how artists and plastic surgeons see what is and what could be.

Artists and plastic surgeons have *ways of seeing what is,* which we have equated to observational ability, and we described this concept through two terms: environmental awareness and subject sensitivity. From the interviews, we defined environmental awareness as being in tune and attentive to one’s surroundings. The ability to observe, or to see or to look, took practice and over time, the observer learned to be aware of subtleties that the untrained eye would not, and thus, have developed subject sensitivity. Both groups were constantly observing their surroundings and had developed this skill acutely. Visual artists looked to the environment for inspiration and motivation. Plastic surgeons reported observing human features in their daily lives to understand the nuances of such features and the anatomy to replicate normal, reconstruct, or attempt to capture beauty surgically. Artists did the same, but their subject matter could be quite broad, sometimes they were human and for others they were landscapes, animals, or creations from their imagination.

The *ways of seeing what could be* related to how each group saw the future in their mind through intention setting and visualization. Intention meant setting the goals of what the artist or surgeon was trying to achieve. We defined visualization as thinking ahead by picturing the final product and going through the process in one’s mind. This visualization also helped with identifying or anticipating issues ahead of time. It was important to set a goal for the work, even if the process changed the end goal. Another key component of visualization was the ability to think spatially and in three dimensions. Setting the intention allowed for visualization. Artists and surgeons described this as going through the steps of the procedure in their minds, considering one’s choices, and imagining the final product.

### Ways of thinking

Finally, these *ways* were all related to *ways of thinking* which involved critical thinking and creativity. Both artists and surgeons had repeatedly stressed the importance of these two concepts in their interviews. From analysis of the coded transcripts, we defined critical thinking as synonymous with problem or puzzle solving along with finding creative solutions. It involved constant self-assessment and self-criticism. Both artists and plastic surgeons had an intense inner dialog often involving the question, “Why is it this way?”. The goal of critical thinking was to innovate, create, improve and understand the self and the work. We defined creativity as pushing the limits or boundaries, thinking outside the box, and being original or new by going beyond the normal, average or expected. Creativity was also closely linked with critical thinking and was born from it. In exploring creativity, we also found a tension between being technically skilled while maintaining innovation. We discovered a push and pull between the technical and the creative and how each comes into play during the process of making. Both groups agree that creativity was a key component of mastery for their respective fields. Artists and plastic surgeons used critical thinking to improve their *ways of doing, knowing and seeing*, so that they could ultimately create and be creative.

### Art versus science

Though there are important similarities in the “Ways of Making” for artists and plastic surgeons, we discovered through the in vivo code “Art vs. Science” an important difference in self-expression versus patient safety. We have demonstrated that plastic surgery is connected to art, but it still originates from the medical sciences. Though there were more similarities than not, the tension between art versus science made it clear that there are boundaries to using artistic concepts in plastic surgery, namely, around the form of self-expression. To describe art, artists and plastic surgeons used words such as “emotion”, “self-expression”, and “right brain”. To describe science, both groups used words such as “logic,” “measurement,” and “left brain.” Science in plastic surgery relates to the medicine of the field; its complications related to patient safety, anatomy, physiology, and human tissue. Art is described as a form of self-expression, where science is not. For example, in visual arts, one can play, paint, or sculpt the human body in unnatural ways and push the human body to extremes as the artists Pablo Picasso and Egon Schiele do. Plastic surgeons cannot because human safety is a high priority in plastic surgery where in art, it is not.

## Discussion

Using a methodologically rigorous approach, we have constructed a model to illustrate how art and plastic surgery share concepts and skills. Our model incorporates data from interviews with practicing visual artists in addition to the perspectives of plastic surgeons. This model is a framework that underpins the work and practices of plastic surgeons and artists and illustrates how they are intimately connected. Our discussion will review the similarity and differences with existing literature, new insights, and impact on curriculum.

Our data are consistent with the current literature that report how these two fields are thought to share similar *concepts*, such as aesthetics and proportion, and *skills*, such as anatomic drawing and manipulation of physical objects, required for occupational mastery [2, 6–9]. We add to these similarities by expanding on these foundational concepts and include *ways of doing* and *ways of knowing.* We also go beyond the physical skills by incorporating the *ways of seeing* to which both artists and plastic surgeons subscribe. Finally, we identified *ways of thinking* and specified two key components of this: critical thinking and creativity. Critical thinking for these participants is synonymous with problem-solving and involves self-assessment. We have defined creativity as pushing the limits or boundaries, thinking outside the box, and being original or new by going beyond the normal, average, or expected. Creativity thrives in the face of boundaries, constraints, or rules because it is in pushing those limits that one can be creative. In understanding these “Ways of Making,” specifically *ways of thinking*, we may be able to teach creativity to our trainees which is strongly correlated with mastery.

The potential overlapping concepts and skills have led some surgeons to suggest inclusion of artistic training in standard plastic surgery education [1, 2, 5, 20]. These plastic surgeons believe that artistic training leads to a better understanding of the concepts of symmetry, proportion, and dimensionality and skills such as anatomic drawing and hand–eye coordination [1, 2, 5–8, 20–22]. Two studies specifically investigated the effect of art education on plastic surgery training. In 1969, Craig Gosling, an artist in the Department of Medical Illustration at Indiana University, designed an art course for plastic surgery trainees that included 16 h of drawing, clay modeling, and molding and casting [1]. He and Lewis Thompson, Assistant Director of the Plastic Surgery Section at that same university, found marked improvement in trainees’ concepts of human symmetry and proportion and better descriptions of patients and communication about surgical techniques. In 2005, another plastic surgeon, Ethem Guneron, emphasized the importance of art education within the field of plastic surgery because of the joint emphasis on “proportion, line, balance, harmony” [21]. With the help of the Department of Art Education, he designed an art course for plastic surgery trainees at Ondokuz Mayis University in Turkey [21]. The curriculum included six hours of general art courses and three hands-on sessions with clay sculpture. After taking the classes, the authors observed that the trainees had improved in the following areas: medical charting of anatomical problems, predicting the result of a surgery based on preoperative markings, and applying more thorough and detailed surgical dressings [21]. Guneron concluded that the art classes helped with overall attention-to-detail. He did not include how these improvements were measured. Furthermore, the authors did not measure improvement in surgical technique, though they concluded that trainees should continue art projects because practice leads to improvement, and trainees should use the learned art concepts to “clearly define esthetic surgical problems” [21].

Both studies could benefit from the model we have presented by aligning their curricular goals with the levels on the model and ensuring that the shared concepts of the model are incorporated. For example, the courses could include a fundamental or foundational lesson that teaches the *elements of art* and *principles of design* and how artists learn anatomy. Another lesson could focus on manual dexterity and technical skill as these authors have designed in the clay and sculpture classes. Any lesson or course would also emphasize the importance of visualization, purposeful action, environmental awareness and subject sensitivity. As an example, this could be done through observations of paintings or even descriptions of human features. Finally, incorporating a component of creativity and problem-solving would be necessary as this represents the highest level on the model. This can be achieved through developing an art practice that is related to plastic surgery.

Other studies have also stressed the importance of art and humanities in medical education [22–24]. Though these studies included a broad range of topics beyond visual arts such as creative writing and philosophy, Moniz et al. developed a *prism model* based off Dennhardt’s previous findings to describe four beneficial epistemic functions of arts and humanities [22, 24]. These are: mastering skills, perspective taking, personal insight and social advocacy [24]. Moniz et al. describe an inability to report a typology of skills due to lack of coherence and broadness of the term in the arts and humanities [24]. Our study, however, which is focused on the visual arts, was able to delineate and describe why technical skills are important. Because Moniz and Denhardt’s models are applicable to the broader world of medical education and arts and humanities, there are differences with our model. Where we describe the importance of goal-setting through visualization and environmental awareness, they describe *perspective taking* as “engaging in art…to facilitate students to relate to and reflect upon their and others’ experiences and thus improve the doctor-patient relationship” [22]. Our model does not include aspects of social advocacy. However, the *prism model* and Denhardt’s model do not include how arts and humanities enhances creativity and critical thinking skills, where it is extremely important in our model and may be more specific to plastic surgery and correlated with obtaining mastery.

The limitations of the study are that the surgeons mostly came from one geographic area that might influence their practice. The artists were more varied in their location. However, many participants in both groups did receive training in classes or programs across the country. Another limitation is that the interviewers were untrained in the visual arts and thus potentially probed differently than experienced artists would. This may have been an asset since this helped in the development of a model with less bias. Further work is needed to explore how the concept model is relevant to other disciplines such as general surgery and head and neck surgery.

## Conclusion

This study provides a model for how plastic surgery and art overlap using data from interviews. Though there are differences between the two fields, the *ways of doing, knowing, seeing, and thinking* are key components of the artistic and surgical processes. This model of “Ways of Making” may offer guidance for educators in plastic surgery to enhance curricula.

## Data Availability

The datasets generated during and/or analyzed during the current study are available from the corresponding author on reasonable request.
